# Lower microhardness along with less heterogeneous mineralization in the femoral neck of individuals with type 2 diabetes mellitus indicates higher fracture risk

**DOI:** 10.1093/jbmrpl/ziae005

**Published:** 2024-03-07

**Authors:** Aleksandar Cirovic, Felix N Schmidt, Marko Vujacic, Praveer Sihota, Bojan Petrovic, Vladimir Zivkovic, Zoran Bascarevic, Slobodan Nikolic, Danijela Djonic, Marija Djuric, Björn Busse, Petar Milovanovic

**Affiliations:** Center of Bone Biology, Institute of Anatomy, University of Belgrade - Faculty of Medicine, 11000 Belgrade, Serbia; Department of Osteology and Biomechanics, University Medical Center Hamburg-Eppendorf, 22529 Hamburg, Germany; Interdisciplinary Competence Center for Interface Research (ICCIR), 20246 Hamburg, Germany; Institute for Orthopedic Surgery “Banjica”; University of Belgrade - Faculty of Medicine, 11000 Belgrade, Serbia; Department of Osteology and Biomechanics, University Medical Center Hamburg-Eppendorf, 22529 Hamburg, Germany; Institute for Orthopedic Surgery “Banjica”; University of Belgrade - Faculty of Medicine, 11000 Belgrade, Serbia; Center of Bone Biology, Institute of Anatomy, University of Belgrade - Faculty of Medicine, 11000 Belgrade, Serbia; Institute of Forensic Medicine, University of Belgrade - Faculty of Medicine, 11000 Belgrade, Serbia; Institute for Orthopedic Surgery “Banjica”; University of Belgrade - Faculty of Medicine, 11000 Belgrade, Serbia; Center of Bone Biology, Institute of Anatomy, University of Belgrade - Faculty of Medicine, 11000 Belgrade, Serbia; Institute of Forensic Medicine, University of Belgrade - Faculty of Medicine, 11000 Belgrade, Serbia; Center of Bone Biology, Institute of Anatomy, University of Belgrade - Faculty of Medicine, 11000 Belgrade, Serbia; Center of Bone Biology, Institute of Anatomy, University of Belgrade - Faculty of Medicine, 11000 Belgrade, Serbia; Department of Osteology and Biomechanics, University Medical Center Hamburg-Eppendorf, 22529 Hamburg, Germany; Interdisciplinary Competence Center for Interface Research (ICCIR), 20246 Hamburg, Germany; Center of Bone Biology, Institute of Anatomy, University of Belgrade - Faculty of Medicine, 11000 Belgrade, Serbia

**Keywords:** type 2 diabetes mellitus, vascular complications, bone mineralization, microhardness, osteocyte lacunar density

## Abstract

There is still limited understanding of the microstructural reasons for the higher susceptibility to fractures in individuals with type 2 diabetes mellitus (T2DM). In this study, we examined bone mineralization, osteocyte lacunar parameters, and microhardness of the femoral neck trabeculae in 18 individuals with T2DM who sustained low-energy fracture (T2DMFx: 78 ± 7 years, 15 women and 3 men) and 20 controls (74 ± 7 years, 16 women and 4 men). Femoral necks of the T2DMFx subjects were obtained at a tertiary orthopedic hospital, while those of the controls were collected at autopsy. T2DMFx individuals had lower trabecular microhardness (*P* = .023) and mineralization heterogeneity (*P* = .001), and a tendency to a lower bone area with mineralization above 95th percentile (*P* = .058) than the controls. There were no significant intergroup differences in the numbers of osteocyte lacunae per bone area, mineralized lacunae per bone area, and total lacunae per bone area (each *P* > .05). After dividing the T2DMFx group based on the presence of vascular complications (VD) to T2DMFx_VD_ (VD present) and T2DMFx_NVD_ (VD absent), we observed that microhardness was particularly reduced in the T2DMFx_VD_ group (vs. control group, *P* = .02), while mineralization heterogeneity was significantly reduced in both T2DMFx subgroups (T2DMFx_NVD_ vs. control, *P* = .002; T2DMFx_VD_ vs. control, *P* = .038). The observed changes in mineralization and microhardness may contribute to the increased hip fracture susceptibility in individuals with T2DM.

## Graphical Abstract

**Graphical Abstract:**
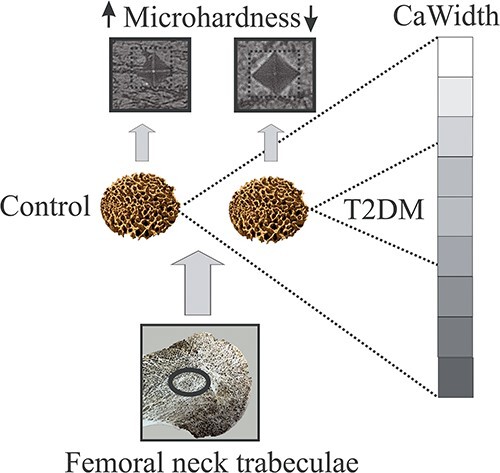

## Introduction

In 2017, it was estimated that 6.28% of the world’s population had diabetes mellitus (DM), and over one million deaths were associated with DM.^1^ If current trends continue, it is expected that >10% of the world’s population will have DM by 2030.^2^ Poor glycemic control is associated with accelerated development of diabetic complications,^3^ which further reduces quality of life^4^^,^^5^ and increases treatment expenses. More than half of the costs for medical treatment are spent on managing diabetic complications.^6^ The mortality rate in patients with hip fracture is higher in those with coexisting DM compared to non-DM patients.^7–9^

In the absence of a better tool, dual-energy X-ray absorptiometry is most commonly used in clinical practice to assess individual risk of fracture. However, many studies have emphasized that individuals with type 2 DM (T2DM) frequently have higher bone mineral density (BMD)^10–13^ than controls; however, T2DM patients are still at an increased risk for sustaining a hip fracture.^8^^,^^14–19^ Many studies have confirmed an increased risk of fracture in individuals with DM,^20–22^ especially of the hip, foot, and spine.^22^ A study conducted on 5285 women with T2DM and 88 120 postmenopausal women without DM showed a 20% higher risk for sustaining any fracture in women with T2DM.^22^ Although the basis for the increased fracture risk in DM individuals has frequently been evaluated, the reported results are often conflicting and no firm conclusion has yet been reached. For example, it has been reported that bone microarchitecture in DM subjects may be worse, equally good, or even better compared with that in non-DM subjects.^23^^,^^24^ Based on the results of microindentation testing (microhardness)/bone material strength index (BMSi), bone material properties may be deteriorated in T2DM individuals compared with controls,^25^ but it is not always the case.^23^ Higher levels of advanced glycation end products (AGEs) are associated with the increased fracture risk in DM subjects, but AGEs levels could also be comparable between DM subjects and controls.^26^ Recently, we have shown lower femoral neck microhardness in T2DM subjects in both compartments (cortical and trabecular)^25^; however, this study was conducted on a small sample size and did not consider other important indicators of bone strength. Sihota et al.^27^ investigated femoral head trabecular bone in patients with DM and fragility hip fractures and reported reduced postyield energy and toughness at the meso-scale in the DM group compared to the non-DM group. Indeed, aside from bone microarchitecture, meso- and micro-scale mechanical properties, and AGEs, other relevant bone characteristics have not yet been sufficiently evaluated in subjects with T2DM, especially at the femoral neck. These include the degree of bone matrix mineralization and osteocyte lacunar parameters. As individuals age, osteocyte lacunar density decreases,^28^ while the proportion of mineralized osteocyte lacunae increases.^29^ Since elderly men and women, in particular, are prone to fractures, alterations in osteocyte lacunar density due to T2DM may be a factor contributing to the fracture risk. In type 1 DM, Kolibová et al.^30^ have recently found an increased number of fully mineralized osteocyte lacunae per bone area in the periosteal region of the cortex of the femoral diaphysis compared to controls. Lower mean matrix mineralization and higher mineralization heterogeneity have been found in vertebrae of non-DM individuals who sustained a fracture.^31^ The analysis of the external cortical bone surface at the superolateral femoral neck of a non-DM hip fracture group revealed a shift to higher mineralization and less heterogeneous mineralization compared with a nonfracture group.^32^ Wölfel et al.^33^ analyzed the midshaft of the femur in T2DM subjects postmortem and found lower osteon density and lower mineralization level with higher heterogeneity in the endocortical and periosteal regions in a fraction of T2DM subjects (those with high cortical porosity). Finally, it is important to consider that recent studies have shown that individuals with T2DM who have developed vascular complications may experience a decline in certain aspects of bone quality, specifically microarchitecture^23^; however, further investigation is required to fully understand this relationship, in particular with respect to other aspects of bone strength besides microarchitecture.

In this study, we analyzed several indicators of bone strength in the trabecular compartment of the femoral neck. Specifically, osteocyte lacunar density, bone matrix mineralization, and bone material properties (microhardness) were examined in individuals with T2DM who experienced fragility fractures (T2DMFx) and controls. In the next step, we compared the measured bone parameters between the T2DMFx subjects with and without vascular complications, and the control group, to examine whether the presence of these complications may influence osteocyte lacunar density, bone mineralization characteristics, and microhardness.

## Materials and methods

### Study design

Femoral neck samples were obtained at a tertiary-level orthopedic university hospital (Institute for Orthopedic Surgery “Banjica,” Belgrade) from 18 T2DM subjects (15 women and 3 men; 78 ± 7 years) who had sustained unilateral low-energy femoral neck fracture. The samples were characterized in detail in Cirovic et al.^34^ Specifically, all of the cases had a prior diagnosis of T2DM and the fracture that resulted from a low-energy trauma; surgical treatment was deemed necessary; and informed consent was provided. The included individuals did not have any known conditions that affect physiological metabolism in bones such as autoimmune and inflammatory diseases (e.g., rheumatoid arthritis), inborn skeletal anomalies, Paget’s disease of bone, chronic liver disease, endocrine diseases such as acromegaly, hypo- or hyperparathyroidism, renal diseases, or any type of malignant tumor.

Control group specimens were obtained postmortem in collaboration with the Institute of Forensic Medicine, Faculty of Medicine, University of Belgrade, from 20 subjects (74 ± 7 years, 16 women and 4 men). These individuals experienced sudden traumatic injuries as the cause of death. They did not have either type 1 DM or T2DM and have never sustained a hip fracture, whether intracapsular or extracapsular. They did not have a history of cancer, renal diseases, primary hyperparathyroidism, Paget’s disease of bone, or any other indications or symptoms of bone disease. Moreover, none of the cases and controls had received medication that could interfere with bone metabolism or impact bone tissue.

We conducted the analyses on the T2DM bone samples and some of the control specimens previously described in Cirovic et al.^34^ The T2DM individuals exhibited a relatively good glycemic control, with an average HbA1c of 6% (minimum 4.3%, maximum 8.4%).^34^ For classifying the T2DMFx subjects to those with and those without vascular complications or vascular diseases (VD), we collected the information regarding the presence of vascular complications from medical history records and autopsy reports.^34^ Moreover, the absence of other disorders that affect bone health was ascertained based on the medical records and autopsy reports, depending on the group.

### Microindentation testing

To evaluate the micromechanical properties of the femoral neck trabecular bone, we utilized a Vickers microhardness tester (HMV-G version, Schimadzu, Japan). The measurements were carried out in line with the previously established conditions for microhardness measurements.^25^ A load of 50 g and an indentation time of 12 s were applied to various trabecular regions. Prior to the microindentation testing, all samples were embedded in resin and polished. Using a 40× magnifying lens, we conducted five separate and valid measurements of the trabecular compartment in each specimen, and the average values were utilized for intergroup comparisons. To avoid boundary effects, we ensured that each indentation was carried out at different trabeculae.^35^ The measurements were carried out independently by two researchers, and the average value was used for the analysis.

### Bone mineral density distribution and osteocyte lacunar density

To examine the bone mineral density distribution (BMDD) and perform a 2D morphological analysis of osteocyte lacunae, quantitative backscattered electron imaging was utilized. The resin-embedded specimens underwent coplanar grinding and were subsequently polished and carbon-coated before imaging. A scanning electron microscope (Crossbeam 340, GeminiSEM, Zeiss AG, Oberkochen, Germany) was operated in backscattered electron mode at 20 keV with a constant working distance of 20 mm. To control the current beam, a Faraday cup was utilized, and grayscale values were calibrated using an aluminum–carbon standard. During imaging, all parameters were monitored and kept at a constant level. Images were acquired at a magnification of 100× (pixel size approximately 2 μm). Four to five images per specimen were captured for the assessment of BMDD (mean calcium weight percentage (mean Ca, wt%); most frequent calcium weight percentage (peak Ca, wt%); SD of the calcium content curve, displaying the heterogeneity of mineralization content across bone area (CaWidth, wt%); percentage of bone area mineralized below the 5th percentile of the reference range of the control group (Ca low, % bone area); percentage of bone area containing calcium concentration above the 95th percentile of the control group (Ca high, % bone area) and osteocyte lacunar morphology analysis. The parameters of BMDD were evaluated from the backscattered electron images using a custom-made MATLAB code (MATLAB, Natick, MA). The size of each image used for osteocyte density analysis was 2.1 mm × 1.6 mm. On the same images, we manually evaluated the following parameters: number of mineralized lacunae per bone area (Mn.Lc.N/B.Ar), number of nonmineralized lacunae per bone area (Lc.N/B.Ar), and total number of lacunae per bone area (Tot-Lc.N/B.Ar) using ImageJ/Fiji (J.53 t; Java 1.8.0_345(64-bit).

### Statistical analysis

The Kolmogorov–Smirnov test was used to verify that the measured parameters in all groups complied with normal distribution. The *t* test for independent samples was used to check for differences in age, body mass index (BMI), parameters of BMDD, osteocyte lacunar parameters (Lc.N/B.Ar, Mn.Lc.N/B.Ar, Tot-Lc.N/B.Ar), and microhardness between the investigated groups (T2DM and controls). In the next stage, one-way ANOVA was performed to check for overall differences in the parameters among the T2DMFx subgroups and control group; when overall ANOVA showed *P*-value < .05, pairwise comparisons (post-hoc tests) under Bonferroni correction for multiple testing were conducted. All analyses were performed two-tailed in SPSS software ver. 15 (IBM SPSS Statistics) at the significance level of 0.05.

## Results

T2DMFx and control subjects did not differ in age (*P* = .113) or BMI (*P* = .292). Moreover, age and BMI did not vary between T2DMFx_VD_ and T2DMFx_NVD_ groups and controls (*P* = .058, *P* = .449). Eight subjects from the T2DMFx group had developed vascular complications (T2DMFx_VD_), while 10 subjects had not (T2DMFx_NVD_), as we have reported in our previous publication.^34^ The median duration of diabetes was 7 years  (1–15), specifically 13 (10–15) years in the individuals with T2DM who had developed vascular complications and 3 (1–9) in the individuals with T2DM and no vascular complications.^34^

**Figure 1:**
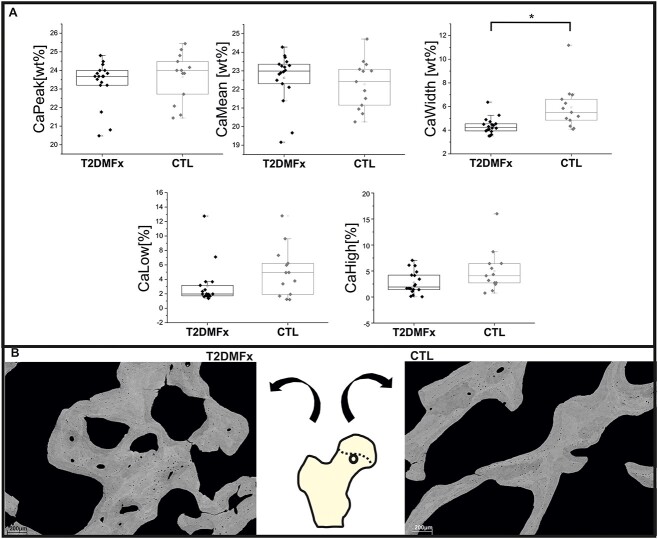
(A) Bone mineral density distribution parameters in T2DMFx subjects and controls. ^*^P < .05. (B) Representative qBEI images of femoral neck trabeculae in T2DMFx subjects and controls.

**Figure 2:**
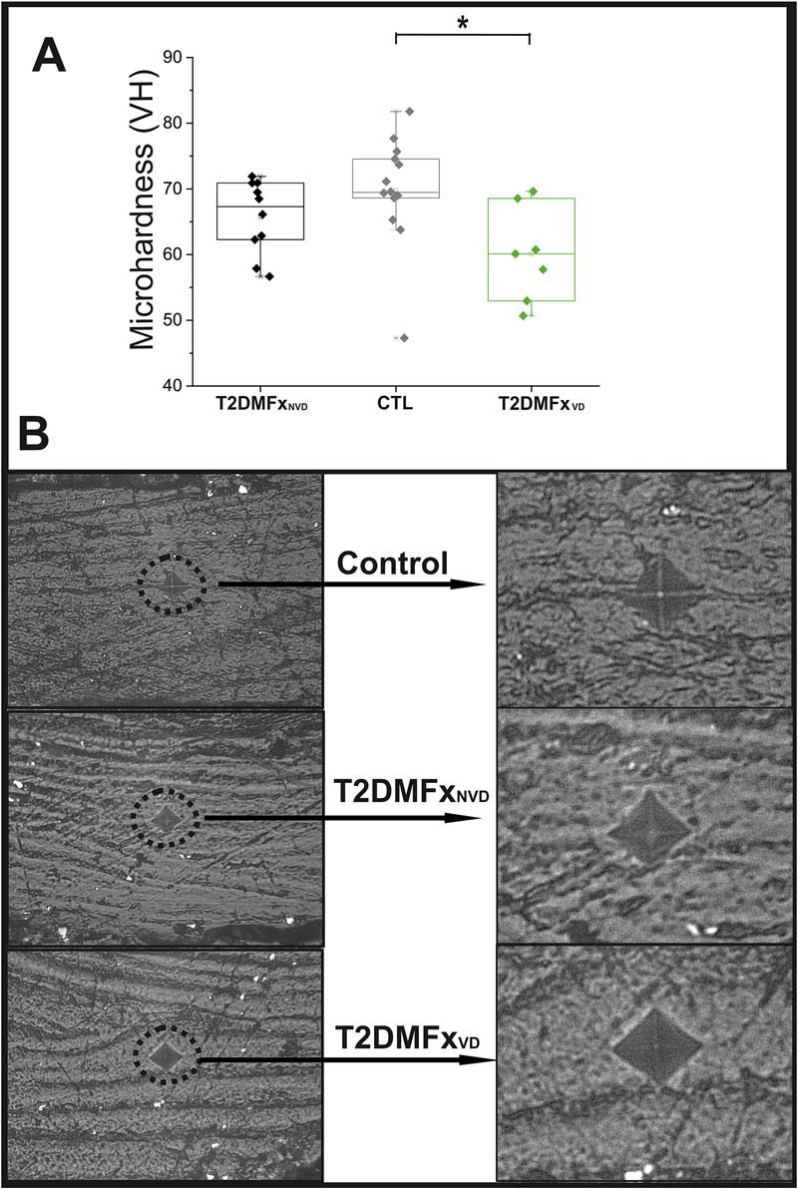
(A) Results of microhardness measurement between three subgroups (T2DMFxNVD, T2DMFxVD, and CTL); ^*^P < .05. (B) Representative images obtained during Vickers microhardness measurement.

BMDD was analyzed in 18 T2DM cases and 13 control cases. There were no significant differences in mean Ca (*P* = .474), peak Ca (*P* = .502), and Ca low (*P* = .092) between T2DM and controls. CaWidth was significantly higher in the controls (*P* = .001), while Ca high was lower in T2DM subjects, but the significance level was not reached (*P* = .058) (Figure 1). After dividing the T2DMFx subjects into the T2DMFx_VD_ and T2DMFx_NVD_ subgroups, we observed significant variations in CaWidth among the three examined groups (*P* = .002). Specifically, CaWidth was significantly lower in the T2DMFx_NVD_ compared with the control group (*P* = .002) and in the T2DMFx_VD_ compared with the control group (*P* = .038), while there were no significant differences in CaWidth between the two T2DMFx subgroups (*P* = 1) (Figures 1 and 2).

Osteocyte lacunar parameters were examined in 18 T2DM cases and 13 control cases. Tot-Lc.N/B.Ar, Mn.Lc.N/B.Ar, and Lc.N/B.Ar did not show significant differences between the T2DMFx and control groups (all *P* > .05). Even after separating the T2DMFx cases to the T2DMFx_VD_ group and the T2DMFx_NVD_ group, no significant intergroup differences were found for any of the osteocyte lacunar parameters (all *P* > .05) (Table 1).

**Table 1:** Osteocyte lacunar parameters of the femoral neck trabecular bone in T2DMFx and control groups.

Parameter	Groups	*n*	Mean	SD	*P*
Lc.N/B.Ar (#/mm^2^)	T2DMFx	18	95.72	12.27	*P* = .382
	Controls	13	91.07	16.91	
Mn.Lc.N/B.Ar (#/mm^2^)	T2DMFx	18	33.51	7.92	*P* = .534
	Controls	13	35.48	9.46	
Tot-Lc.N/B.Ar (#/mm^2^)	T2DMFx	18	128.95	15.31	*P* = .570
	Controls	13	125.4	19.08	

Vickers microhardness was analyzed in 17 T2DM cases and 14 control cases. The control specimens had higher trabecular bone microhardness than T2DMFx specimens (*P* = .023). After distinguishing the two groups of T2DM individuals based on the presence of VD, one-way ANOVA showed that trabecular bone microhardness still varied among the control, T2DMFx_VD_, and T2DMFx_NVD_ groups (*P* = .023), but the difference was only significant between the T2DMFx_VD_ and control groups (*P* = .02). Microhardness was similar between the T2DMFx_VD_ and T2DMFx_NVD_ groups (*P* = .353) and between the T2DMFx_NVD_ and control groups (*P* = .563) (Figure 2).

## Discussion

In this study, we examined BMDD parameters, density of osteocyte lacunae, and microhardness of the trabecular bone within the femoral neck of T2DMFx subjects and controls. We found lower microhardness and lower CaWidth in the femoral neck trabecular bone, indicating that T2DM may induce changes in the bone matrix mineralization characteristics, leading to a decreased mechanical bone resistance. Moreover, within the T2DMFx group, we were able to identify differences in femoral neck trabecular microhardness; namely, T2DMFx_VD_ subjects had lower microhardness compared with the T2DMFx_NVD_. Given that no significant differences were found in mean Ca, reduced microhardness cannot be explained exclusively by the changes in mineralization pattern. This means that there may be some changes in organic components of the bone matrix, which requires further studies. In principle, such expectations may be supported by a previous study that has shown that Vickers microhardness is reduced after bone samples are treated by collagen-altering treatments (boiling, NaOCl treatment).^36^

We found lower total osteocyte lacunar density than reported in previous studies,^28^^,^^29^^,^^37^ which could be explained by the differences in the bone compartment and skeletal site analyzed. Specifically, cortical bone has higher osteocyte density than trabecular bone,^28^ and there is substantial heterogeneity in osteocyte lacunar density between different skeletal sites.^28^^,^^29^^,^^37^

In our study, we found a lower CaWidth in individuals with DM and fractures. In a multiscale assessment of T2DM-induced bone changes, Wölfel et al. examined the mineralization profile of the cortex of the femoral diaphysis in three groups of individuals postmortem: subjects with T2DM and normal porosity, T2DM subjects with pronounced cortical porosity, and control cases. They found higher CaWidth in the endocortical region of T2DM individuals with pronounced cortical porosity.^33^ Although the results reported by Wölfel et al. are in contrast with those from the present study, there are two main differences. First, Wölfel et al. analyzed the femoral diaphysis cross-sections, and second, the focus of the study was on the cortical bone, which naturally has higher CaWidth when compared to trabecular bone,^38^ whereas our study focused on the trabecular bone. Furthermore, Wölfel et al. did not specifically examine fracture samples like we did in the present study. Pritchard et al.^39^ also evaluated the degree of mineralization in T2DM individuals. They collected femoral neck specimens obtained after surgery due to osteoarthritis and analyzed the trabecular compartment. The authors found higher mean calcium and lower CaWidth in T2DM individuals and suggested that this could be explained by altered bone remodeling.^39^ However, all examined subjects had osteoarthritis; hence, it is not possible to distinguish the effects of T2DM, and to what extent OA causes changes in BMDD parameters. Yet, a more homogeneously mineralized bone material in the fractured DM cases in our study may be more prone to fracture due to a less effective fracture energy dissipation.^40–42^ Misof et al. analyzed bone mineralization in transiliac bone biopsy specimens obtained from 26 young (pre-menopausal) women. In that study, neither cancellous nor cortical BMDD parameters from these individuals were significantly different from reference BMDD values.^43^ However, that study only included premenopausal women and assessed specimens from the iliac bone.

Previously, we^44^ analyzed various aspects of BMDD in the femoral diaphysis of 51 female donors postmortem; similar values of CaWidth were found between young and osteoporosis groups, while aged individuals had lower CaWidth compared with both groups. Moreover, apart from BMDD parameters, we performed an osteocyte lacunar density evaluation based on the qBEI images and found that aged subjects had significantly lower Lc.N/B.Ar and higher Mn.Lc.N/B.Ar compared with young subjects. In contrast, individuals with osteoporosis did not differ from controls in either of these parameters. We did not find differences between the two groups with respect to the osteocyte analysis, which is in line with our earlier data^44^ since the investiagted groups did not differ in age. Taking BMDD and osteocyte lacunar density evaluation into consideration, it is reasonable to speculate that T2DM and osteoporosis have adverse effects on bone quality but in a different manner, but these aspects warrant future investigation T2DM subjects more commonly experience hip and humerus fractures, while wrist fractures are less common among individuals with DM but very common in patients suffering from osteoporosis.^45^^,^^46^ It is possible that T2DM accelerates bone aging, as evidenced by AGEs accumulation in bone tissue.^40^ Lower CaWidth indicates more homogeneous mineralization, which causes relatively uniform mechanical properties and facilitates crack propagation in the shortest trajectory. In case of higher heterogeneity of bone mineralization, a number of interfaces are formed, and they may deflect and guide crack path, thereby leading to a gradual dissipation of fracture energy and preventing a complete fracture.^47^

Previously, we have measured the microhardness of the superolateral femoral neck’s cortical and trabecular compartments in eight subjects with diabetes and eight controls and showed lower microhardness^25^ in both compartments of the T2DM subjects. The present study represents a significant step forward in better understanding of the mechanisms involved in the increased hip fracture risk in T2DM individuals by analyzing the T2DM cases who sustained a hip fracture. Similarly, Sihota et al.^27^ reported decreased nanoindentation-derived modulus and hardness in the femoral head trabecular bone of 30 subjects with DM and 40 non-DM individuals with fragility hip fractures. Farr et al. reported a lower BMSi at the tibia mid-shaft in 30 T2DM women compared with non-DM controls. Nonetheless, Samakkarnthai et al. found no difference in BMSi at the same site in a larger cohort that included 171 T2DM patients and 108 age-matched non-DM subjects of both sexes.^23^ Holloway-Kew also determined BMSi using OsteoProbe in 340 men (234 controls, 59 subjects with impaired fasting glucose, and 47 T2DM subjects). When all three groups were statistically analyzed with ANCOVA, no differences were observed^48^; however, when the authors fused controls with subjects with impaired fasting glucose as a non-DM group and compared them with T2DM individuals, a significant difference appeared, i.e., T2DM subjects showed a significantly lower BMSi. The main advantage of our present study and our previous study^25^ is that we examined microhardness of the area more relevant for a typical fracture in T2DM, while all three other studies focused on the tibia, which is not frequently fractured in T2DM patients.^45^

When separating DM cases into those with and those without VD, we did find a lower microhardness in those with VD. Previously, several studies have reported significant bone effects of vascular complications in individuals with T2DM,^23^^,^^34^ but these were limited to the effects on microarchitecture. Specifically, we highlighted the detrimental impact of vascular complications on the femoral neck trabecular microarchitecture in T2DMFx subjects,^34^ and Samakkarnthai et al.^23^ demonstrated worsened cortical microarchitecture in the distal tibia of T2DM subjects with vascular complications. Considering both these studies alongside our current findings, it becomes evident that the presence of vascular complications can affect various aspects of bone material properties, including microarchitecture, microhardness, and CaWidth. Consequently, further investigation is warranted to fully understand the implications of vascular complications on bone health (in T2DM individuals) in the future. Of note, in contrast to microhardness, CaWidth was not much affected by the presence of vascular complications given that there were no significant differences between the T2DMFx_VD_ and T2DMFx_NVD_ subgroups, but both groups showed a significant reduction in CaWidth compared with the control group. This means that T2DM itself has some effects on bone mineralization, independent of vascular complications.

There are certain limitations that need to be acknowledged. First, this research was conducted in a cross-sectional study design, which has inherent limitations in determining causality. Second, it is important to note that all cases of T2DM in this study were treated. This conflation of effects makes it challenging to discern the distinct impact of T2DM from that of the treatment on the outcomes of interest. Nevertheless, most of the individuals received the same oral antidiabetic medication (metformin in 14/18 individuals, sulfonylureas in 8/18 individuals), and the distribution of used medications was the same in both T2DM subgroups. Third, the decision to conduct surgical treatment in hip fracture cases was based on various factors, including the overall patient condition and glycemic control; therefore, the range of HbA1c values in the T2DM group in this study may not be representative of the entire T2DM population. Fourth, bone turnover markers and hormone levels were not routinely analyzed in the orthopedic hospital (T2DM group), and biochemical analysis was not meaningful in autopsy cases (control group).

## Conclusion

We have observed several impairments in femoral neck trabecular bone induced by T2DM. First, CaWidth was lower in T2DMFx subjects, indicating less heterogeneous mineralization distribution of the trabecular bone. Second, trabecular microhardness was lower in T2DMFx subjects, and the presence of vascular complications particularly contributed to a decrease in trabecular microhardness. These demonstrated alterations in trabecular structure represent part of the overall changes that diabetes can induce and that were shown by other studies. The mentioned trabecular impairments could lead to the increased risk of fracture during a sideways fall.

## Data Availability

The data that support the findings of this study are available from the corresponding authors [P.M.] and [B.B.] upon reasonable request.
